# Minutes to hours after a nuclear event: available radiation poisoning antidotes and practical considerations on possible urgent approaches

**DOI:** 10.1007/s00259-023-06305-1

**Published:** 2023-06-27

**Authors:** Xhoajda Taci, Giulia Poletto, Flavio Trotti, Fabiana Gramegna, Alessandra Zorz, Chiara Giraudo, Francesca Venturini, Flavio Seno, Nicola Realdon, Roberto Vettor, Sonia Faoro, Diego Cecchin

**Affiliations:** 1 Hospital Pharmacy Unit, Padova University Hospital, Padua, Italy; 2 Nuclear Medicine Unit, Department of Medicine (DIMED), Padova University Hospital, Padua, Italy; 3 ARPAV Unità Organizzativa Agenti Fisici, Verona, Italy; 4 Istituto Nazionale Di Fisica Nucleare, Laboratori Nazionali Di Legnaro, Legnaro (Pd), Italy; 5 Department of Medical Physics, Veneto Institute of Oncology IOV – IRCCS, Padua, Italy; 6 Department of Physic and Astronomy (DFA), Galileo Galilei”, INFN Section, University of Padova, Padua, Italy; 7 Department of Pharmaceutical and Pharmacological Sciences, University of Padova, Padua, Italy; 8 Department of Medicine (DIMED), Padova University Hospital, Padua, Italy

## Introduction: nuclear emergencies and possible scenarios

A nuclear emergency is a type of accident that can expose a highly variable number of people to isotopes and radiation [1]. The worst-case scenario involves the *detonation of a military nuclear weapon*. The radioactive fallout is very complex, and the main threat is the exposure to external radiation.

Among the other possible causes of nuclear disasters, *accidents or acts of sabotage at nuclear power plants* like the [2, 3] explosions in the reactors in Chernobyl (1986) and Fukushima (2011) led to the release of large amounts of radioactive substances into the atmosphere, with severe consequences, especially for the population living closest to the power plant, and for the environment [1, 3]. The most important routes of contamination were external irradiation from deposition on the ground and ingestion, followed by inhalation and irradiation due to the passage of the “radioactive cloud.” If the radionuclides ingested were I-131 Cs-137 and Cs-134; then, the spectrum of isotopes inhaled broadens, mainly to include Ru-103, Te-132, I-131, Cs-134, Cs-136, Cs-137, Ba-140, Ru-106, and Ce-141 [4]. The dose to which individuals might be exposed depends largely on the urban conditions and environmental characteristics of the area affected at the time of the accident. National emergency management plans [5] could, for example, consider the location of the nuclear power plant to identify possible scenarios: plants up to 200 km from the country’s border (the plan envisages possible iodo-prophylaxis [5, 6], sheltering indoors, and food restrictions); those more than 200 km from the border (preventive measures, such as food restrictions, and protecting agricultural products and livestock); and plants in non-European countries (no protective measures are suggested).

Nuclear emergencies can also be caused by the *detonation of radiological dispersion devices* (RDDs) loaded with isotopes, also known as “dirty bombs” [7]. In this case, the types of radioactive substance that can enter the atmosphere/air and could be inhaled are more limited (probably just one, because fission does not occur). The impact of such a nuclear emergency is hard to estimate but predictive analyses suggest that the numbers of people involved would be fairly small, with a relatively low health risk related to radioactive fallout [8]. Although numerous different isotopes can be used in RDDs, the options can be reasonably narrowed down to the nine most widely available: americium-241 (^241^Am), californium-252 (^252^Cf), cesium-137 (^137^Cs), cobalt-60 (^60^Co), iridium-192 (^192^Ir), plutonium-238 (^238^Pu), polonium-210 (^210^Po), radium-226 (^226^Ra), and strontium-90 (^90^Sr) [9].

*Malicious nuclear contamination of the environment* is another possibility to consider. In this case, the critical factor is the time elapsing between the dispersion of the radioactive material and the contamination of the population. An example of such a scenario is the Goiania accident that took place in Brazil in the 1980s, when two men took a source of cesium-137 from an abandoned clinic and sold it in parts. The symptoms of radiation poisoning in the parties involved were not recognized immediately, and this led to their inadequate treatment and allowed the contamination to spread [10].

Lastly, the *poisoning* of Alexander Litvinenko on November 2006 [11] using polonium-210 has brought into attention also the possibility of a direct contamination of drinking water or food.

The above-described scenarios naturally differ considerably, but some isotopic contaminants are more common than others. It is therefore important to know what antidotes are available, and in what dosages they should be used. In this editorial, we first present a non-exhaustive, practical list of the antidotes available (for the adult population) for the most common isotopic contaminants (Table 1). Then, we examine which antidotes with a safe profile (available, in many countries, without the need of a medical prescription) are available for use in an urgent intervention to deal with a large-scale nuclear event.

**Table 1:**
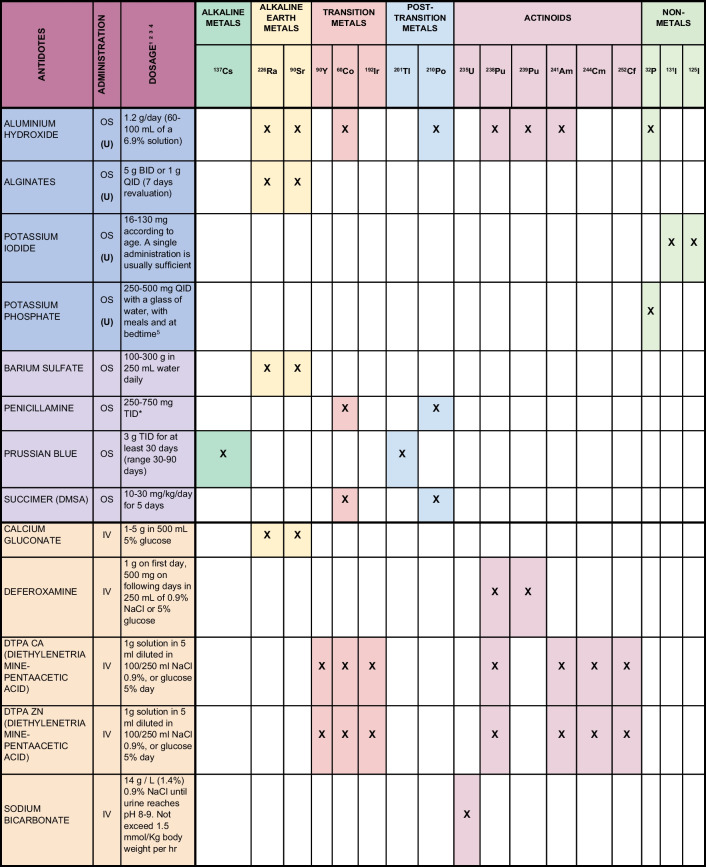
Antidotes potentially suitable (in adult population) in a number of isotopic contaminations together with their mode of administration and suggested dosages

Sources: (1) IAEA_Medical Management of Persons Internally Contaminated with Radionuclides in a Nuclear or Radiological Emergency. 1 March 2019; (2) Micromedex; (3) Antidotes/Drugs data sheets (when available); (4) WHO. National stockpiles for radiological and nuclear emergencies: policy advice. ISBN 978–92-4–006787-5. (5) Domínguez-Gadea L, Cerezo L. Decontamination of radioisotopes. Rep Pract Oncol Radiother J Gt Cancer Cent Poznan Pol Soc Radiat Oncol. 7 July 2011;16(4):147–52

Legend: *OS* orally administered, *IV* intra-venously administered, *(U)* possible use in an urgent approach. *The dosage here reported for penicillamine refers to the poisoning with copper, lead, gold, iron, mercury, and other heavy metal reported in “IAEA_Medical Management of Persons Internally Contaminated with Radionuclides in a Nuclear or Radiological Emergency.”

Please note: Interactions between different antidotes have not been considered in the paper and in Table 1. We here assume the use of a single antidote for a specific isotope in adult population (pediatric dosages have not been considered)

It should be stressed out that this work deals with the medical management of people involved in a nuclear emergency, as such, it is mainly addressed to the medical personnel appointed for treatment decisions; antidotes should be used, in any case, only after medical/officer advice.

## Available antidotes for internal decontamination

Exposure to radionuclides can lead to two types of contamination, external and internal. The major health risks examined here concern internal exposure to radiation, which occurs generally through inhalation, ingestion, and absorption through the skin, wounds, or burns. The primary goal of internal decontamination is to prevent absorption and increase excretion of the radioisotopes. Decontamination is more effective the sooner it is initiated: once the tissues have incorporated the isotopes, the situation is virtually irreversible. The damage caused by the biological effects of ionizing radiation can lead to deterministic or stochastic effects such as, for example, acute radiation syndrome (ARS), and/or long-term carcinogenic effects [10].

Acute radiation sickness occurs within hours or days after exposure, and its severity is related directly to the dose of radiation involved. The most common symptoms include skin rashes, dermatitis, anemia and leukopenia, and mucosal hemorrhages in the most severe cases. For the onset of ARS, it has been estimated that the absorbed dose must reach at least 0.5–1.0 Gy [12].

The occurrence of long-term effects is presumably more likely the higher the dose of radiation absorbed, but it is impossible to establish a threshold below which they can be ruled out. The severity of these long-term effects is also not dose-dependent, and they are described as stochastic because they are difficult to predict and influenced by a number of variables [13]. The most common outcomes of a stochastic effect of internal contamination are various forms of cancer, leukemia, and genetic defects because the radiation damages the structure of nucleic acids [14]. To limit such stochastic effects, it is therefore important to prevent the incorporation and deposition of radionuclides in target organs as much as possible. The molecules used for this purpose act mostly through the following mechanisms: inhibiting gastrointestinal absorption with the aid of sequestering agents, displacing the radionuclide by administering its non-radioactive counterpart, forming complexes with chelating agents, and ingesting substances that stimulate the radionuclides’ excretion [10]. The antidotes used for decorporation therapy are mostly administered off-label. Most of these treatments have not been authorized by the regulatory agencies for this specific therapeutic indication. Prussian blue, potassium iodide (KI), calcium-diethylenetriaminepentaacetic acid (Ca-DTPA), and zinc-diethylenetriaminepentaacetic acid (Zn-DTPA) are the only antidotes authorized for decontamination purposes: Prussian blue for radioactive cesium; KI for iodine; and Ca- or Zn-DTPA for plutonium, americium, or curium. As clinical trials would be unthinkable for ethical reasons, the available safety and efficacy data come mainly from experience gained in the field after nuclear accidents and from animal studies [15]. Considering the most common sources of contamination and the available international guidelines and scientific literature, we identified a list of antidotes potentially suitable (in adult population) for widespread use. These antidotes are listed in Table 1, together with their mode of administration, and suggested dosages. The “cocktail” use of antidotes in patients simultaneously contaminated with multiple isotopes [16] is a complex issue that goes beyond the aim of this paper. The reader should however be aware that one antidote could counteract the efficacy of another one (such as for example bicarbonate for uranium + DTPA for plutonium) or even present harmful side effects if combined.

## Urgent and precautionary approaches

To ascertain the possible absorbed dose of radiation, and the most appropriate antidote, it is essential to establish the radionuclide involved, but this can take time—especially in the case of alpha emitters [17]. Unfortunately, time is a decisive factor in efforts to ensure the most effective decontamination possible, and delaying the start of treatment can be harmful to the people contaminated. With the exception of stable iodine (where WHO/IAEA recommendations on timing [6, 10] are available), there is no consensus on when to start treatment with other antidotes, and whether or not to wait for internal dosimetry [17]. While timely administration makes an antidote more effective, its use should always have a favorable risk–benefit ratio.

There are other important aspects to consider when it comes to preventive campaigns for a general population that may or may not have been affected by the nuclear accident, including the antidote’s safety, availability, and ease of administration. In fact, the various strategies generally adopted to manage a nuclear event can currently be divided into two categories: the precautionary approach and the urgent approach. The precautionary approach provides treatment in relation to the committed effective dose and the isotope involved. The urgent approach, in cases of suspected contamination, delivers treatment (after specific isotopes in the environment have been identified, at least, but without the need for any “a priori” individual internal dosimetry) [18–20] within few hours [17]. We can assume that an urgent approach could be considered for the general population (for people closer to the event, at least) and that the easier-to-use antidotes are more appropriate in this scenario (delaying the internal dosimetry evaluation to whenever possible). The precautionary approach could be more reliable for individuals more directly affected by the nuclear event and more suitable especially when the antidotes to administer require hospitalization. Antidotes with a more complex safety profile that can only be administered intravenously or that are not yet available in large quantities should be reserved for people known to be contaminated or directly involved in the accident, and therefore more likely to have absorbed high doses of radiation. In short, for large-scale nuclear events, the urgent approach probably achieves better medical outcomes (due to a speedier antidote administration), while the precautionary approach could be safer [21] and less costly (at least in terms of antidotes) but with the added risk of a loss in effectiveness (due to a delayed administration).

It is probably wise to administer the few, safe, and economical antidotes according to an urgent approach (without any individual dosimetry) after detecting specific radioisotopes in the environment to the population closest to the event, at least (at highest risk of contamination). This would enable a larger number of people to be managed immediately, and therefore more effectively, and ensure an albeit partial decontamination. The exact area to cover would depend on the specific scenario and on environmental variables. This approach does not preclude a proper individual dosimetric evaluation and a subsequent shift to a more adequate antidote if necessary. Among the antidotes discussed in the literature, those possibly eligible for urgent, large-scale use in the general population closest to the nuclear event are adsorptive agents, such as aluminum hydroxide and sodium alginate, often available as over-the-counter products; potassium phosphate, available as a food supplement; and KI because of its time-dependent mechanism of action. In the following paragraphs, we review the available literature on these agents. All the other antidotes, many of which have to be administered intravenously, have a much more complex pharmacological (and safety) profile. They are consequently much less suitable for use according to an urgent approach. An exhaustive review of the literature on these latter types of antidotes goes beyond the aims of this paper.

## Antidotes available for large-scale use according to an urgent approach

1. *Adsorptive agents: alginates and aluminum hydroxide*

*Alginates*, normally in the form of sodium or calcium salts, are commonly used to treat gastroesophageal reflux. They are available in various pharmaceutical formulations, from granules to chewable tablets to oral suspensions. After ingestion, they form a highly viscous gel that can also bind alkaline earth metals such as strontium, calcium, barium, and radium. They are consequently recommended, off label, to prevent the absorption of radionuclides. The suggested dosage ranges from 500 mg twice a day (BID) to 1000 mg four times a day (QID) for 7 days, after which a clinical assessment is recommended [10]. Several studies, in both preclinical and clinical settings, have been conducted to validate the use of alginates for preventing strontium (Sr) uptake. The studies on humans (conducted on volunteers) all showed that alginates can reduce Sr uptake [22–24]. Some authors reported a potential risk of a concomitant reduction in the absorption of trace elements, such as calcium (Ca^2+^) [22–24]. Guidelines for intervention after a nuclear disaster do not mention any increase in calcium excretion as a side-effect of alginates, however. Alginates are therefore considered safe, with practically no side-effects in most cases, and could be an appropriate choice for large-scale urgent interventions. According to the International Atomic Energy Agency (IAEA), major adverse effects may rarely occur in patients on low-salt diets or in diabetics given alginate tablets containing a certain amount of sugar [10]. These risks could be quickly checked before administering the antidote, however.

*Aluminum hydroxide* is used as an antacid because, combined with the hydrochloric acid produced by the gastric mucosa, it lowers the acidity of the stomach's contents, relieving the symptoms of conditions like gastritis, gastric ulcer, and gastroesophageal reflux. IAEA guidelines suggest the off-label use of this agent in cases of gastrointestinal (GI) contamination with a broad spectrum of radionuclides because it is able to sequester the isotope, preventing its absorption and promoting its excretion [10]. The recommended dose of aluminum hydroxide for this indication is 1–2 g/day. For some radionuclides (cobalt, polonium, and strontium), aluminum hydroxide is clearly indicated by the IAEA to manage emergencies, while for others (americium and plutonium), its use is only suggested. Despite the recommendations in the guidelines, it is hard to find published studies supporting the use of aluminum hydroxide in cases of radionuclide contamination especially in a large population. According to a study conducted on healthy volunteers by Bingham et al. [25], aluminum hydroxide can prevent the absorption of phosphorus from food (though the dose needed to do so is unclear). Since the pharmacokinetic properties of the isotope are similar to those of the native element, we can assume that aluminum hydroxide can prevent the absorption of ^32^P as well, though there is nothing in the literature to confirm this hypothesis. Like alginates, aluminum hydroxide is not associated with any major adverse reactions. It is often found in pharmaceutical formulations that also contain magnesium hydroxide to counterbalance the astringent effect of the former with the laxative effect of the latter. Given the safety profile of these drugs and their potential beneficial effect in internal contamination, although presenting a low scientific evidence, they could both seem suitable for use in a large-scale urgent approach to a nuclear event.

2) *Potassium phosphate*

Phosphorus 32 has been used in nuclear medicine, for both therapeutic and diagnostic purposes in oncology. Accidental exposure to this radionuclide can require decorporation action, however. Guidelines recommend the oral ingestion of 250–500 mg of potassium phosphate four times a day, with meals and at bedtime, to block radioactive phosphorus uptake. The ingestion of potassium phosphate is sometimes contraindicated, however, mainly in cases of hyperphosphatemia and moderate-to-severe renal impairment. There have been rare reports of mild gastrointestinal symptoms, such as abdominal heaviness, nausea, and (occasionally) diarrhea during the treatment [7, 10, 26]. Potassium phosphate is available as a food supplement and therefore readily available to the general population. It has a good safety profile. That said, there are no published studies of any kind on the use of potassium phosphate to treat internal contamination from radioactive phosphate, the duration of such treatments, or the strength of the evidence of their efficacy.

3) *Potassium iodide*

Contrary to the other drugs presented (adsorptive agents and potassium phosphate) so far, potassium iodide is a well-known and specific drug used as “antidote” to block thyroid uptake of radioiodine. We decided to include a brief paragraph on potassium iodide for the sake of completeness but we recommend World Health Organization [6] and IAEA [10] publications for a complete deepening of the topic (which is beyond the scope of this paper). Iodine and cesium were identified as the main contaminating radionuclides after the Chernobyl and Fukushima accidents [27]. The incidence of thyroid cancer consequently rose in the areas surrounding Chernobyl (Belarus, Ukraine, and the western part of the Russian Federation). The main source of iodine radioisotopes is from uranium fission processes. When a nuclear accident occurs, the dispersion of these isotopes can cause internal contamination [28]. Among the 14 radioisotopes deriving from iodine, the main ones are iodine 125 and iodine 131, which is also used in diagnostics. About 25–30% of the iodine 131 absorbed by the body accumulates in the thyroid, while the rest is generally quickly excreted with the feces or urine [10]. The radionuclides absorbed by the thyroid could lead to thyroid dysfunction and thyroid cancer. Children and adolescents are particularly prone to this type of damage due to a greater sensitivity of the tissues affected in the young [28]. KI is the agent indicated to limit the accumulation of radio-iodine in the thyroid. Ingesting stable KI enables serum concentrations to exceed those of the radioactive isotope. This leads to a saturation of the thyroid and speeds up the elimination of the remaining circulating iodide (radioactive and non-radioactive). For this mechanism to develop and be as effective as possible, the stable KI needs to be ingested as soon as possible after the presumed contamination (within 4 h). Administered after more than 24 h, it even becomes harmful as it may prolong the elimination of the radioiodine that has already accumulated. The suggested dose depends on the clearance of iodine (and its isotopes), which varies with age. A single dose of 130 mg of KI is recommended for adults and adolescents over 12 years old (although repeated administration may be necessary after prolonged or repeated exposure). Its use is contraindicated in cases of hypersensitivity to iodine, Hashimoto’s thyroiditis, Basedow’s disease, other autoimmune thyroid diseases, low-complement vasculitis, and herpetiform dermatitis. The risk of adverse events after the oral ingestion of KI is low (5 × 10^−7^) although at the dose of 100–150 mg/die, it could induce hypothyroidism in the newborns and hyperthyroidism in the elderly. The most likely reactions are hypersensitivity and, in cases of a prolonged thyroid uptake blockade, a reduced metabolic activity and gland hypertrophy [10]. KI should be given primarily to infants, nursing mothers and children because they are at greatest risk [29]. Following the release of radioactive iodine due to the Chernobyl accident, the Polish government launched a campaign to administer KI to the general population. A subsequent efficacy assessment found that children given KI one to four days after exposure had an approximately 50% lower committed dose to the thyroid [30]. Some evidence has been collected from epidemiological studies, but no data deriving from clinical studies are available. The World Health Organization (WHO) conducted a systematic review to examine whether administering stable iodine for thyroid blockade affects the risk of developing thyroid cancer, hypothyroidism, or benign thyroid nodules. Of the 2177 studies identified, only 4 were considered eligible and found a correlation between KI intake in children and a lower risk of thyroid cancer [29]. In a further systematic review, Pfinder et al. [28] confirmed—with a low to very low quality of evidence—that taking KI after a nuclear accident may reduce the risk of thyroid cancer in children. No comparable data are available regarding the use of KI by adults. The FDA has authorized its use for internal radioiodine contamination in the form of tablets or a solution (iOSAT tablets, 130 mg, from Anbex, Inc.; ThyroSafe tablets, 65 mg, from BTG International, Inc.; Potassium Iodide Oral Solution USP, 65 mg/mL, from Mission Pharmacal Company). The FDA also recommends its use primarily in children and lactating women and secondarily (and for higher exposures) in adults aged 18 to 40 years. Beyond the age of 40, people are at lower risk of encountering the stochastic effects of exposure to iodine isotopes, so the administration of KI should be assessed on a case-by-case basis [29, 31]. The tablets can be crushed and taken with fruit juice, jam, milk, or the like. This antidote is normally available in a hospital setting but, in an emergency, it could be distributed to the general population with attached safety instructions.

## Conclusions

Multiple scenarios can arise following a nuclear event, and it is hard to say what actions might be applicable to all of them. Intervention should be adapted to the radionuclides involved, but unfortunately, this information is not available before the disaster occurs.

Based on international guidelines, we have discussed the recommended antidotes for the main radionuclides associated with a nuclear emergency. Unfortunately, most of these treatments have not been approved by the regulatory authorities, and the suggested doses do not derive from studies on their efficacy for decontamination purposes. At least we can assume that their toxicity has been assessed, as these substances are used in humans for other indications (Table 1). In addition to the choice of drug, another important aspect is the interval elapsing between the contamination and the start of treatment.

The type of treatment required is closely related to the nature of the radionuclide (making dosimetric evaluations mandatory), but it is fundamentally important to start the treatment as soon as possible. These two aspects are difficult to reconcile. It can take time to identify radionuclides deposited in the environment and to measure the dose adsorbed by a given individual (which decides the need for specific treatments), but treatments must be started promptly to avoid target organ contamination and deposition. This raises the quandary of whether treatment should be preventive (the urgent approach), or only administered after contamination has been confirmed by individual dosimetric checks (the precautionary approach) [21, 32–34].

We looked at readily-available antidotes with a very safe pharmacological profile that could be administered prophylactically to reduce the risk of incorporation in the general population (at least for people closest to the event) once an isotope has been detected in the environment. We focused on drug treatments that do not require hospitalization. These criteria restricted the field to alginates, aluminum hydroxide, and potassium phosphate. We also, for the sake of completeness, considered potassium iodide.

Aluminum hydroxides seem suitable for a noninvasive intervention that can do no harm. Even the IAEA guidelines recommend oral aluminum hydroxides to inhibit the gastrointestinal absorption of several radionuclides (Am, Co, P, Pu, Po, and Sr) [10]. This indication probably stems from their non-specific ability to inhibit gastrointestinal absorption as no studies have specifically examined their efficacy for decorporation. Internal contamination by americium and cobalt derives from their gastric absorption. Only a minimal part of americium is absorbed, but it persists in the organism (mainly liver and bones) for many years. On the other hand, 10–30% of ingested cobalt goes beyond the gastrointestinal barrier; about 5% is deposited in the liver, accumulating in vitamin B12 molecules, but it has an estimated biological half-life of 6 days [9]. Like americium, plutonium reaches the bloodstream only minimally through the gastrointestinal tract. It is more commonly absorbed through inhalation and deposited in the liver and bones, where it persists for a long time. The fraction of polonium absorbed after ingestion varies considerably; it then becomes unevenly distributed with about 45% depositing in the spleen, kidneys, and liver, and about 10% in the bone marrow [9].

Alginates are believed to inhibit the intestinal absorption of alkaline earth metals like strontium, calcium, barium, and radium [10]. Studies in volunteers that focused on inhibiting strontium uptake demonstrated an effective reduction in its systemic absorption, with a presumably lower accumulation in the bones as a consequence [22–24]. Approximately 30–40% of ingested strontium reaches the systemic circulation and its metabolism is similar to that of calcium: 31% of strontium in the bloodstream is deposited in the bones, where residues are found up to a year later. Radium behaves in much the same way and is deposited mainly in the bones and teeth. The radium contamination risk is greater after inhalation whereas most ingested radium (80%) is eliminated with the feces [9]. For all these radioisotopes, there are other antidotes, even of first choice, that would be preferable to antacids, but most of them would require intravenous administration and/or strict medical supervision, so they could only be made available to the individuals most severely affected by the nuclear event, not to the general population.

Phosphorus-32 tends not to be included in the lists of the most likely radionuclides released in a nuclear emergency [35]. If ingested, 60% is excreted within 24 h, while the remainder probably follows the metabolic pathway of non-radioactive phosphorus, the main target being the bones [35]. Given this assumption regarding its biodistribution, some guidelines recommend administering potassium phosphate in cases of suspected contamination: it would compete with the radioactive isotope and thereby limit its absorption. The risk of accidental phosphorus-32 contamination in the general population is low, and no studies have confirmed the efficacy of potassium phosphate. We have nonetheless included this antidote in our discussion because of its excellent safety profile in terms of toxicity. We believe that the benefits of the treatment would outweigh the risks in the event of a suspected contamination.

Inhaled or ingested iodine radioisotopes have a very high permeability [9]. Once in the circulation, they accumulate mainly in the thyroid. KI should be administered as soon as possible to avoid internal contamination, in younger patients at least, as its ability to saturate the thyroid gland declines if the treatment is delayed [10]. The use of KI is supported by the regulatory authorities and already envisaged by government bodies for the large-scale management of a nuclear emergency [17, 36].

Coping with a nuclear emergency is a complex process. There are many factors to consider, and the impact of the event can never be fully predicted, but a clinical response to the needs of the population will always be required. Antidotes for radioisotope decontamination vary in their mechanisms of action and safety profiles. Decisions regarding the most appropriate treatments should take into account not only clinical but also managerial and economic aspects. Antidotes such as KI are already indicated for a priori urgent general administration. Judging from our analysis, other antidotes with an extremely favorable safety profile, such as aluminum hydroxide, could also be candidates for an urgent approach to large-scale nuclear events.

More systematic and evidence-based studies are needed, however, to support guidelines on the antidotes to administer, both to the general population and to the individuals most directly affected, in the event of a nuclear emergency.
